# *Aspergillus* Endocarditis: A Rare but Serious Complication During Treatment With Ibrutinib

**DOI:** 10.1155/crdi/6863015

**Published:** 2025-09-25

**Authors:** Luca Mezzadri, Ilaria Giuseppina Chiara Caramma, Irene Maria Sciabica, Guglielmo Marco Migliorino, Annalisa Cavallero, Giovanni Marchetto, Giuseppe Lapadula, Paolo Bonfanti

**Affiliations:** ^1^Infectious Diseases Unit, Fondazione IRCCS San Gerardo dei Tintori, Monza, Italy; ^2^School of Medicine and Surgery, University of Milano-Biccoca, Milan, Italy; ^3^Microbiology Unit, Fondazione IRCCS San Gerardo dei Tintori, Monza, Italy; ^4^Cardiac Surgery Unit, Fondazione IRCCS San Gerardo dei Tintori, Monza, Italy

**Keywords:** *Aspergillus* endocarditis, case report, fungal endocarditis, ibrutinib, immunocompromised host

## Abstract

**Background:** *Aspergillus* endocarditis (AE) is a rare but life-threatening form of infective endocarditis, accounting for only 0.2% of cases. Despite antifungal and surgical treatment, AE remains a major diagnostic and therapeutic challenge with high mortality rates. Ibrutinib, a Bruton's tyrosine kinase inhibitor used in the treatment of chronic lymphocytic leukemia (CLL), has been associated with early-onset invasive aspergillosis. However, no cases of AE have been documented in patients undergoing ibrutinib therapy to date.

**Case Presentation:** A 71-year-old man with relapsed CLL on third-line ibrutinib therapy and a history of arrhythmic cardiomyopathy requiring an implantable cardioverter-defibrillator (ICD) presented with a six-month history of fever, cough, and fatigue. On admission, a full-body computed tomography scan revealed intestinal ischemia and multiple thrombotic occlusions involving the kidney, spleen, and iliac artery. Markedly elevated beta-D-glucan and serum galactomannan levels prompted empirical initiation of isavuconazole. Transthoracic echocardiography identified a large vegetation (2.5 × 4 cm) on the mitral valve with ICD lead involvement. Despite urgent surgical intervention—including ICD extraction and mitral valve replacement—the patient succumbed to complications. Intraoperative valve cultures grew *Aspergillus fumigatus.*

**Conclusions:** This report underscores the severity of AE despite combined surgical and antifungal therapy. Given its high mortality rates, clinicians should maintain a high index of suspicion for AE, particularly in immunocompromised patients or those with a history of cardiac surgery or implanted cardiac devices. Early recognition and aggressive intervention remain essential to improving outcomes.

## 1. Introduction

Fungal endocarditis constitutes approximately 2%–4% of all cases of endocarditis, with *Aspergillus* being the second most common pathogen following *Candida* species [1].

*Aspergillus* endocarditis (AE) remains a rare cause of infective endocarditis, accounting for only 0.2% of cases [2].

Significant risk factors for its development include previous cardiothoracic surgery and profound immunosuppression, particularly in patients with hematological malignancies or those undergoing solid organ transplantation (SOT) [2, 3]. Additionally, ibrutinib, a Bruton's tyrosine kinase (BTK) inhibitor used in the treatment of various hematological conditions, including chronic lymphocytic leukemia (CLL), has been associated with early-onset invasive aspergillosis (IA) [4]. Among IA cases, cardiac involvement may occur, leading to AE.

The diagnosis and management of AE present significant clinical challenges due to the often subtle presentation. Despite surgical and antifungal therapy, the mortality rate remains exceedingly high, ranging from 50% to 95% [5].

Here we present a particularly challenging case of *Aspergillus *native-valve endocarditis with involvement of the implantable cardioverter-defibrillator (ICD) in an immunocompromised patient undergoing ibrutinib therapy.

## 2. Case Description

A 71-year-old Caucasian male presented to the emergency department with a six-month history of fever, cough, and fatigue. His medical history was notable for CLL diagnosed 14 years earlier, requiring multiple treatment regimens due to disease relapses. Eighteen months before the current presentation, he was treated at another hospital for pulmonary aspergillosis with isavuconazole, started at that time for a suspected but unconfirmed concomitant mucormycosis. Six months prior to his current presentation, he began third-line treatment with ibrutinib for relapse of CLL. Approximately two months after starting ibrutinib, he developed COVID-19 pneumonia, requiring hospitalization and leading to discontinuation of the medication. He also had a history of hemolytic anemia, treated 6 months earlier with high-dose steroids due to a relapse. Additionally, he had an ICD placed 22 years earlier following a ventricular fibrillation episode, with periodic device interrogations confirming proper device function, including a cardiology visit 2 months before the current presentation.

Despite being discharged after the episode of COVID-19 pneumonia, the patient remained SARS-CoV-2 positive with persistent low-grade fever, for which he received multiple antiviral treatments without complete resolution of symptoms.

Upon admission to our hospital, he exhibited a low-grade fever, and a holosystolic murmur was audible over the mitral area, radiating to the axilla, consistent with mitral regurgitation. The SARS-CoV-2 nasal swab was still positive. Laboratory assessments revealed anemia, leukopenia, and thrombocytopenia. Multiple sets of blood cultures were negative. Bronchoalveolar lavage (BAL) revealed no evidence of acid-fast bacilli, and galactomannan (GM) antigen testing (VirClia MonoTest, Granada, Spain; cutoff value < 0.16) was negative. A multiplex panel for bacterial and viral pathogens performed on BAL returned negative results. Serological tests for *Brucella* and *Bartonella*, as well as blood polymerase chain reaction (PCR) testing for adenovirus, *Enterovirus*, herpesvirus, *Cytomegalovirus*, and Epstein–Barr virus, all yielded negative results.

**Figure 1:**
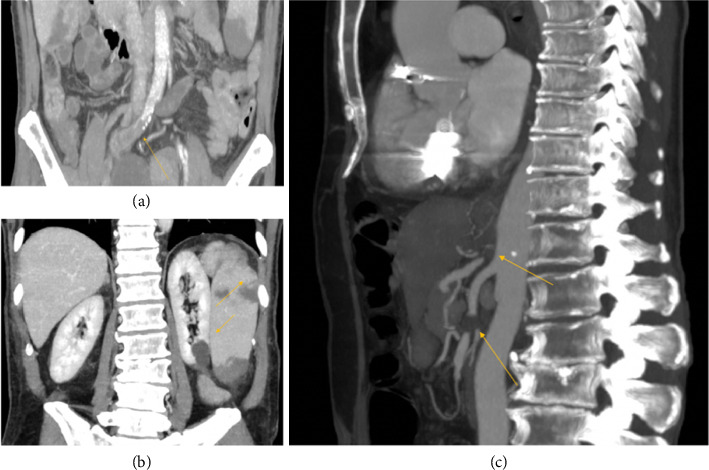
Computed tomography findings. (a) Occlusive thrombus of the external iliac artery (arrow). (b) Thrombotic occlusions of the left kidney and spleen (arrows). (c) Occlusive thrombus in the proximal superior mesenteric artery and at the origin of the celiac trunk (arrows).

A full-body computed tomography (CT) scan revealed an occlusive thrombus of the external iliac artery (Figure 1(a)), as well as thrombotic occlusions involving the left kidney and spleen (Figure 1(b)), and an occlusive thrombus in the proximal segment of the superior mesenteric artery and at the origin of the celiac trunk (Figure 1(c)).

**Figure 2:**
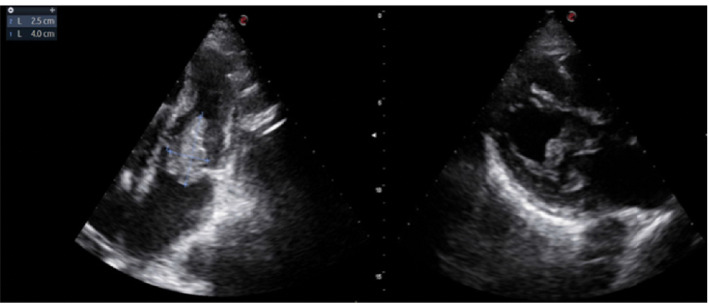
Transthoracic echocardiogram showing 2.5 × 4 cm mitral valve vegetation.

Beta-D-glucan serum levels of 1310 pg/mL (FUJIFILM Wako Chemicals Europe GmbH, Neuss, Germany; cutoff value≤ 7 pg/mL) and a serum GM antigen index of 2.37, without recent exposure to common causes of false-positive results such as piperacillin–tazobactam, albumin, intravenous immunoglobulins, or blood transfusions, prompted the empirical initiation of intravenous isavuconazole (200 mg per day after loading doses). In this setting, isavuconazole was preferred over voriconazole for its more predictable pharmacokinetics, easier management, lower risk of QTc prolongation and drug–drug interactions, and previous use in this patient for pulmonary aspergillosis. A transthoracic echocardiogram (TTE) was performed, revealing a vegetation on the ICD lead and a large mitral valve vegetation measuring 2.5 × 4 cm (Figure 2). Given the need for urgent surgery and the risk of hemodynamic compromise, the TTE findings were considered sufficient for diagnosis, and a transesophageal echocardiogram was not performed.

During hospitalization, the patient experienced abnormal ICD shocks, suggesting device malfunction. Electrophysiological analysis confirmed the malfunction, necessitating intensive care unit transfer for continuous rhythm monitoring. Following a multidisciplinary evaluation, the patient underwent a combined surgical intervention, including ICD replacement and simultaneous cardiac valve replacement on day 4 from the endocarditis diagnosis. Intraoperatively, the ICD leads were released from adhesions, the generator was extracted, a fracture of one of the leads at the level of the superior vena cava was noted, and finally complete extraction was achieved. The mitral valve was exposed, revealing extensive and severe damage from a broad endocarditic process with multiple large vegetations infiltrating the subvalvular apparatus and posterior wall. Complete removal of the mitral valve and vegetations was performed, samples were sent for culture, and mitral valve was successfully replaced with a bioprosthetic valve.

Tissue samples were processed in tryptic soy broth (TSB, bioMérieux, France), vortexed, and subsequently inoculated on different aerobic and anaerobic media. ICD lead tips were cultured using the same broth enrichment procedure, without the use of sonication, which is not routinely available in our laboratory. Histopathological examination of the valve tissue was not performed.

Isavuconazole therapy was maintained until surgery, for a total of 4 days of antifungal treatment. Unfortunately, the patient's condition deteriorated, and he died 24 h after the surgical procedure for refractory septic shock. One day after the patient's death, growth from valve samples was observed after 4 days of incubation on PVX chocolate agar and Sabouraud agar. *Aspergillus fumigatus* was identified based on colony morphology and confirmed by MALDI-TOF mass spectrometry. ICD lead cultures remained negative after 10 days of incubation on all media.

## 3. Discussion

We presented the case of an immunocompromised patient who developed AE with lead infection of ICD after receiving treatment with ibrutinib. To the best of our knowledge, this is the first documented case of AE in a patient receiving ibrutinib treatment.

AE is a rare but well-documented complication in immunocompromised individuals, particularly those with hematologic malignancies and prolonged neutropenia. In a review by Valerio et al., which described 60 definite cases of AE, the primary risk factors for its development in immunocompromised individuals were solid organ transplant or hematopoietic stem cell transplant (HSCT) [2]. Another review found that approximately one-third of patients with AE had hematological malignancies [6].

Although patients with CLL are considered at low risk for developing IA, treatment with tyrosine kinase inhibitors, including ibrutinib, has been increasingly associated with its development [7, 8]. This association is due to ibrutinib's immunosuppressive effects, which disrupt B-cell receptor signaling and alter neutrophil function, both crucial for controlling fungal infections [9]. Recent in vitro experiments have also shown that ibrutinib-induced depletion of BTK impairs signal transduction pathways in macrophages, hindering the effective eradication of *Aspergillus fumigatus* [10]. In this case, the patient had a history of multiple immunosuppressive treatments and infections, with ibrutinib potentially playing an additional role in the pathogenesis of the condition. Moreover, in line with findings from Fürstenau et al. [11], our patient was male, heavily pretreated (≥ 3 previous treatment regimens), and had received corticosteroid therapy, all of which are risk factors for development of invasive fungal infections (IFIs) under ibrutinib.

IA associated with ibrutinib therapy often manifests early, even after a short duration of therapy [12]. According to Gold et al., most IFIs following small molecule kinase inhibitor therapy manifest more than 90 days after treatment initiation [13]. In our case, the patient developed symptoms approximately 2-3 months after starting treatment, complicated by persistent fever potentially related to ongoing COVID-19, which initially misguided the diagnosis of endocarditis symptoms.

The pathogenesis of cardiac involvement in this patient was likely multifactorial. A previous episode of invasive pulmonary aspergillosis may have served as a colonization site for *Aspergillus fumigatus*, enabling hematogenous dissemination in the setting of profound immunosuppression. ICD-related infection could have acted as a nidus for vegetation formation and subsequent mitral valve involvement, although a primary valve infection cannot be excluded.

Furthermore, prolonged SARS-CoV-2 positivity for more than 8 weeks in immunocompromised hosts has been associated with an increased risk of IA [14]. In this patient, persistent PCR positivity likely reflected marked immunosuppression and may have contributed to his susceptibility to disseminated *Aspergillus* infection, together with other risk factors such as prior corticosteroid use and ibrutinib therapy.

The clinical presentation of AE is often nonspecific, posing a significant diagnostic challenge. Our patient had a 6-month history of fever, cough, and fatigue—symptoms that could easily be attributed to other infections or malignancy-related complications. Despite multiple recent hospitalizations, there was no evidence of cardiac involvement at the previous examinations. However, persistent low-grade fever and the identification of occlusive thrombi on a CT scan prompted further investigation, ultimately leading to the diagnosis of endocarditis. Although central nervous system involvement is relatively common in IA among patients receiving ibrutinib, particularly those with primary cerebral lymphoma [12], our patient's brain CT scan showed no pathological findings.

Laboratory findings indicative of fungal infection in our patient included elevated beta-D-glucan and GM levels. The sensitivity of serum GM assays is highly dependent on the patient population being tested. In nonneutropenic patients, GM sensitivity is generally lower compared to other groups, and it drops to around 20% in SOT recipients. However, it tends to be higher in patients with hematological malignancies or those who have undergone allogeneic HSCT [15]. A meta-analysis of over 5000 immunocompromised patients evaluating the diagnostic performance of the Platelia© ELISA test for GM detection found a serum GM sensitivity of 82% and specificity of 81% using a 0.5 optical density (OD) index cutoff [16].

In our patient, blood cultures were negative, a common finding in IA, with only 10% of cases yielding positive results [17]. The definitive diagnosis was achieved only after surgery with *postmortem* results, as subsequent valve culture grew *Aspergillus fumigatus* on the mitral valve, highlighting the difficulty in reaching a definitive diagnosis in this setting. This aligns with previous studies, which found that in approximately one-third of cases, AE was only diagnosed *postmortem*, and fewer than half of patients were diagnosed prior to surgery [18]. Other reported cases have incorporated additional diagnostic approaches, including molecular confirmation through gene sequencing [19].

Although ICD involvement was demonstrated through ultrasound imaging, lead cultures turned negative results. In clinical practice, culture sensitivity for cardiac implantable electronic device (CIED) infections can vary widely, with studies reporting detection rates ranging from approximately 60%–80% when leads are involved [20, 21]. Sensitivity can be influenced by factors such as the method of sample collection and the concentration of organisms within biofilms on the device surface [22]. Sonication has been shown to increase diagnostic yield [23].

Treatment of AE is often challenging and typically requires a combination of surgery and aggressive antifungal therapy. Early surgical intervention is recommended to reduce embolic risk. Based on low-quality evidence derived mainly from case series, case reports, and animal models, clinical practice guidelines suggest voriconazole or liposomal amphotericin B as initial antifungal options, with lifelong therapy following valve surgery [15]. Similarly, recent consensus guidelines from the Australasian Society for Infectious Diseases (ASID) recommend voriconazole as first-line therapy [12]. Isavuconazole, despite being approved as the primary therapy for IA and demonstrating noninferiority to voriconazole for this indication, is not explicitly recommended as a first-line therapy for AE due to the lack of specific data for this clinical presentation. Nonetheless, in our case, it was considered an appropriate alternative, given its more predictable pharmacokinetics, favorable safety profile, and lower risk of QTc prolongation and drug–drug interactions. These aspects were particularly relevant in a critically ill patient with ICD malfunction, and the choice of the drug was further supported by prior use in this patient for pulmonary aspergillosis. Amphotericin B was avoided due its nephrotoxicity, given the patient's renal thrombotic occlusions and the associated risk of kidney failure.

Surgical intervention in AE is generally recommended as early as possible to maximize survival; in our patient, surgery was performed 4 days after diagnosis. Earlier intervention might have improved the prognosis, although the overall clinical severity and multiple complications likely contributed to the poor outcome.

This is consistent with the high mortality rates reported in the literature [2, 5, 18]. These rates approach 100% for patients treated with medical therapy alone [24].

## 4. Conclusion

AE can represent a rare but severe complication in immunocompromised patients undergoing treatment with ibrutinib. This case highlights the importance of early diagnosis and the need to maintain a high clinical suspicion for AE in this population. However, despite medical and surgical treatment, mortality rates remain significantly high.

## Data Availability

All relevant data supporting the findings of this case report are included in the manuscript. Additional details can be provided by the corresponding author upon reasonable request.
